# The Multiple Roles of CD147 in the Development and Progression of Oral Squamous Cell Carcinoma: An Overview

**DOI:** 10.3390/ijms23158336

**Published:** 2022-07-28

**Authors:** Giovanni Barillari, Ombretta Melaiu, Marco Gargari, Silvia Pomella, Roberto Bei, Vincenzo Campanella

**Affiliations:** 1 Department of Clinical Sciences and Translational Medicine, University of Rome Tor Vergata, Via Montpellier, 00133 Rome, Italy; ombretta.melaiu@uniroma2.it (O.M.); marco.gargari@gmail.com (M.G.); silvia.pomella@uniroma2.it (S.P.); bei@med.uniroma2.it (R.B.); vincenzo.campanella@uniroma2.it (V.C.); 2 Department of Hematology and Oncology, Cell and Gene Therapy, Bambino Gesù Children’s Hospital, Viale S. Paolo, 00146 Rome, Italy

**Keywords:** CD147, oral keratinocytes, inflammation, EMT, oral premalignant diseases, oral squamous cell carcinoma, hypoxia, cancer stem cells

## Abstract

Cluster of differentiation (CD)147, also termed extracellular matrix metalloprotease inducer or basigin, is a glycoprotein ubiquitously expressed throughout the human body, the oral cavity included. CD147 actively participates in physiological tissue development or growth and has important roles in reactive processes such as inflammation, immunity, and tissue repair. It is worth noting that deregulated expression and/or activity of CD147 is observed in chronic inflammatory or degenerative diseases, as well as in neoplasms. Among the latter, oral squamous cell carcinoma (OSCC) is characterized by an upregulation of CD147 in both the neoplastic and normal cells constituting the tumor mass. Most interestingly, the expression and/or activity of CD147 gradually increase as healthy oral mucosa becomes inflamed; hyperplastic/dysplastic lesions are then set on, and, eventually, OSCC develops. Based on these findings, here we summarize published studies which evaluate whether CD147 could be employed as a marker to monitor OSCC development and progression. Moreover, we describe CD147-promoted cellular and molecular events which are relevant to oral carcinogenesis, with the aim to provide useful information for assessing whether CD147 may be the target of novel therapeutic approaches directed against OSCC.

## 1. Introduction

Squamous cell carcinoma (SCC) arising from the oral epithelium (oral SCC, OSCC) is an aggressive and metastasizing tumor which accounts for over 90% of oral cavity malignancies: its incidence, which has always been very high in Asia, has also been recently growing in Western Countries, making OSCC the sixth most common human cancer [1,2,3].

At present, OSCC treatment options include surgery, cytotoxic chemotherapy, and external radiotherapy, often in multimodal regimens [4].

In patients with OSCC at an advanced stage of progression, extensive and mutilating surgical interventions are required, which do not prevent tumor recurrence and metastatization [5]. The administration of cisplatin and fluorouracil, together with pembrolizumab, a monoclonal antibody enhancing antitumor immune responses by blocking the programmed cell death protein 1, is the first-line chemotherapy for OSCC [5]. However, 2/3 of patients do not respond adequately to this treatment, or they develop resistance against it, especially when the OSCC is in an advanced stage of progression [5]. Similarly, the efficacy of radiotherapy is also often compromised by the onset of radio resistance in OSCC cells [6]. For all these reasons, the five-year survival rate of advanced stage OSCC patients has not changed in the last 60 years, settling at around 50% [7]. Therefore, the prognosis and the quality of life of OSCC patients largely depend on the timeliness of diagnosis.

In this regard, it should be considered that the onset of an OSCC is, in most cases, preceded by the development of dysplastic and/or hyperplastic lesions, which, taken together, are defined as “oral potentially malignant disorders” (OPMDs) [8]. The etiologic agents of OPMDs are varied and include: (i) bacteria infecting the periodontium; (ii) repeated mechanical traumas (such as, for example, those caused by sharp teeth or incongruous dental prostheses); (iii) tobacco chewing or smoking; (iv) alcohol abuse; (v) hypersensitivity reactions promoted by dental materials; (vi) oncogenic, high-risk human papilloma viruses [5,8,9,10].

Among these OPMDs is leukoplakia, a white plaque which results from a hyperplastic thickening of the superficial layers of the buccal epithelium and abnormal keratinization of the keratinocytes [8]. Leukoplakia often regresses, whereas other OPMDs persist [8]. Among those OPMDs are erythroplakia (a plaque lined with a mucous epithelium), the lichen planus (a chronic inflammatory disease associated with a dysfunction of cellular immunity), and oral submucosal fibrosis (a degenerative process that initially affects the lamina propria of the oral mucosa and then deepens in the submucosa causing loss of elasticity) [8]. While homogeneous leukoplakia and oral lichen planus have a very low risk of malignant evolution, non-homogeneous leukoplakia, erythroplakia, and oral submucosal fibrosis have a high probability of progressing first to a non-invasive carcinoma (“carcinoma in situ”) and then to an invasive OSCC [11]. Generally, an OSCC may arise during the first two years following the detection of an OPMD, although it has been documented that the risk lasts for 10–15 years [8]. Based on this evidence and considering that the oral cavity can be easily inspected, it is important to monitor OPMDs regularly and remove those at high risk [8].

Although the histopathological examination of tissue biopsy remains the most effective procedure to distinguish OPMDs from OSCCs [5,8], the degree of epithelial dysplasia may not always define the risk of OPMD progression to OSCC [12]. Hence, there is an urgent need to find reliable biomarkers allowing for the early diagnosis of OSCC. Likewise, deepening the knowledge of the biomolecular pathways leading to the onset of OPMDs and/or their evolution into OSCCs is essential for designing new therapeutic approaches which could hopefully be more effective than those applied nowadays.

In this context, a large body of studies has evaluated the diagnostic/prognostic value of surveilling key players in the growth, survival, invasion, and differentiation of oral epithelial cells and biological events deregulated in OPMDs and OSCCs [13,14,15,16,17]. Among the markers examined in the aforementioned studies are the basic helix-loop-helix twist homolog (TWIST) transcription factors, the Ki67 cell proliferation marker, the Bcl-2 survival factor, the pro-apoptotic Bax, the protein kinase B (AKT), the pro-invasive matrix metalloproteinases (MMPs), the cell membrane cadherins and connexins, and cytoskeleton components such as keratins or vimentin [13,14,15,16,17]. Further work has focused on the modulators of biological processes that are altered during oral carcinogenesis, such as the remodeling of the extracellular matrix (ECM) [18,19,20,21,22], the metabolism of glucose [23], and the inflammatory response [24,25].

In this regard, it must be highlighted that epithelial cells growth/locomotion, ECM turnover, glycolysis, and inflammation share a feature in common: to be affected by the activity of CD147, a transmembrane glycoprotein belonging to the immunoglobulin superfamily [26]. CD147 is expressed by oral keratinocytes with an intensity that gradually increases as the oral mucosa becomes the site of a reparative process, a chronic inflammatory disease, or a tumor [18,27,28,29,30,31]. Indeed, the overexpression and/or functional deregulation of CD147 have been linked to the development and progression of a large variety of carcinomas [31].

Based on all these findings, herein, we summarized and discussed the results from studies regarding CD147 impact on oral carcinogenesis, which suggests a possible use of CD147 for early monitoring of the risk of OSCC onset and progression. Data were searched for in the PubMed Central electronic database of the National Library of Medicine (National Institutes of Health, Bethesda, Maryland, United States of America). The search was carried out from November 2021 to May 2022 and was updated in July 2022. There was no time restriction on the studies included, and the final data consisted of studies published from 1989 to 2022. In total, 445 articles were screened in accordance with the aim of the review. Approximately, 315 articles were selected for full-text screening, and 240 articles were included in the final study.

The results from the examined studies point at CD147 as a promising novel marker for monitoring the onset and clinical progression of OSCC, as well as a likely target of innovative therapeutic strategies directed against this aggressive tumor.

## 2. CD147 and Invasive OSCC Development

The CD147 protein is either anchored to the surface of many human cell types, oral keratinocytes included, or it is secreted, free, or exosomes-bound [26]. Upon the glycosylation of the asparagine residues of its extracellular domain [32], cell surface anchored CD147 is activated and forms homodimers with another glycosylated CD147 expressed or released by neighboring cells (Table 1) [26]. Alternatively, membrane-bound CD147 is triggered by ligands such as galactoside-binding galectin-3 and cyclophilin A isomerase (Table 1), which are expressed, and often released by most human cell types [26,33,34]. On the other hand, the activity of CD147 is hindered by caveolin-1, a putative tumor suppressor preventing CD147 glycosylation [35].

The actions of CD147 are manifold. Among them, the best-known is its effective participation in the remodeling of the ECM, which occurs during physiologic tissue growth, maturation, and/or repair, but also in pathologic settings such as chronic inflammatory diseases and tumors [26]. CD147’s effects on the ECM are due to CD147’s ability to interact with cell-matrix adhesion receptors, for example, integrins [40,41], and to activate ECM-degrading proteolytic enzymes such as MMPs (Table 2) [26,38,41,42,43,44,45,46].

MMPs are a group of proteases which redundantly cleave ECM components as well as cellular adhesion molecules, cytokines, growth factors, or their receptors [58,59]. It is just from the capability of activating MMPs that another term by which CD147 is called originates, namely extracellular MMP inducer [26].

**Figure 1:**
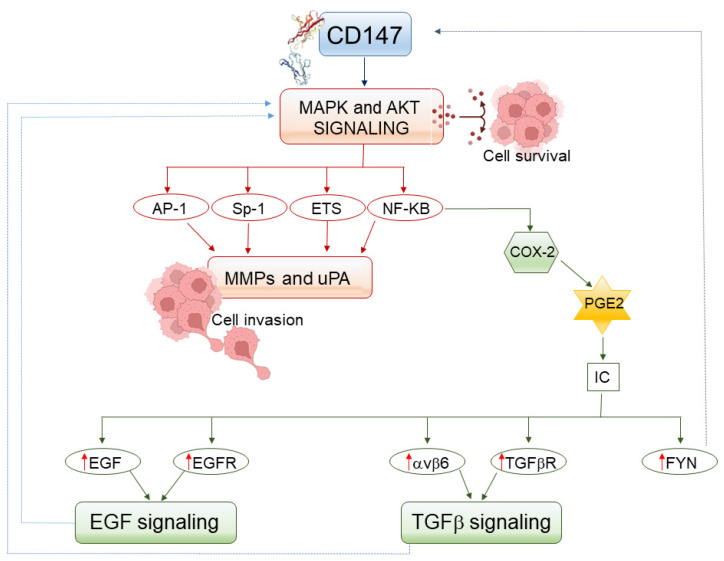
CD147 triggering leads to the expression and/or functional activation of proteolytic enzymes, inflammatory mediators, and growth factors with a key role in oral carcinogenesis. Arrows symbolize directions of connections. Abbreviations: AKT, protein kinase B; AP-1, Activator Protein-1; COX-2, cyclooxygenase-2; EGF, epidermal growth factor; EGFR, epidermal growth factor receptor; IC, inflammatory cytokines; MAPK, mitogen-activated protein kinase; MMP, matrix metalloproteinase; NF-kB, Nuclear Factor-kappa B; PGE2, prostaglandin E2; Sp-1, Specificity protein-1; TGFβ, transforming growth β; TGFβR, transforming growth β receptor; uPA, urokinase-like Plasminogen Activator.

Specifically, the stimulation of CD147 leads to the activation of mitogen-activated protein kinases (MAPK)/extracellular-regulated kinases (ERK) and the phosphoinositide 3 kinase (PI3K)/AKT intracellular signaling pathways [43,60,61,62,63,64,65], which will then turn on transcriptional activators of MMP expression such as Nuclear Factor-kappa B (NF-kB), Activator Protein (AP)-1, Specificity protein (Sp)-1, and E26 transformation-specific (ETS) factor (Figure 1) [66,67,68,69,70,71].

An additional proteolytic enzyme whose synthesis is induced upon CD147 activation is the urokinase-type plasminogen activator (uPA) (Table 2) [48,72,73,74]. The latter can straightly degrade the ECM [75] or convert latent plasminogen to active plasmin that, in turn, digests the ECM both directly and by activating MMPs (Table 2) [76]. As with MMPs, the expression of uPA is also preceded by the activation of MAPK and/or AKT signaling, and can be induced by NF-kB, AP-1, Sp-1, or ETS transcription factors (Figure 1) [77,78,79,80].

In healthy oral mucosa, CD147 is detected mainly on the surface of the cells constituting the basal layer [29]. There, CD147 associates with α3β1 and α6β1, two integrins that contribute to establishing the polarity of epithelial basal cells by binding the laminin constituting the epithelial basement membrane (Table 2) [40,41].

The levels of CD147 expression or activity increase in epithelial cells located at the edge of a wound [28]. There, CD147 triggering is followed by the synthesis of interstitial MMPs, which, in turn, disassemble intercellular adhesions, thereby ending cell contact inhibition and starting the growth and locomotion of epithelial cells, which lead to the closure of wound margins (Table 2) [28].

CD147 is further upregulated in chronically inflamed oral mucosa, where the protein is detected not only in the epithelium but also in the underlying connective, being synthesized by fibroblasts and inflammatory cells [29].

The expression of CD147 is dramatically increased in OPMDs and invasive OSCCs [18,30,31]. Differently from what occurs in healthy oral mucosa, CD147 is detectable throughout the entire OSCC lesion [18,30]. As observed for CD147, CD147 agonists cyclophilin A and galectin-3 are overexpressed in OSCC tissues as compared with healthy oral mucosa, their levels being significantly correlated with OSCC relapse or distant metastasis and poor overall survival of the patients [81,82]. At the same time, the CD147 inhibitor caveolin-1 is frequently downregulated or functionally impaired in OSCC, leading to CD147 hyper glycosylation and, therefore, hyperactivation [41].

As expected, the expression and activity of MMPs increase in OPMD and OSCC tissues in parallel with CD147 hyperactivation [18,19]. A trend opposite to that of MMPs is followed by their antagonists, such as the Tissue Inhibitors of MMPs (TIMPs), whose amounts in OSCC lesions are lower than those found in healthy oral mucosa [83] and inversely related to CD147 expression (Table 2) [47].

In oral lesions, MMPs are synthesized by dysplastic or transformed epithelial cells and by cancer-associated fibroblasts (CAFs) [18,29,30]. Specifically, SCC cells produce MMPs after their highly glycosylated membrane-bound CD147 homodimerizes with another CD147 molecule expressed on the surface of neighbor cells or released by them in the extracellular compartment (Table 1) [26]. Alternatively, the CD147 expressed on the membrane of SCC cells can be triggered by cyclophilin A (Table 1) [33,43]. On their part, CAFs synthesize MMPs following the binding of their CD147 to that present on the OSCC cell membrane or released by OSCC cells (Table 1) [18,29,30].

Among CD147-induced MMPs, MMP-1 mediates the initial phase of OSCC invasion because of its capability of degrading both cell-to-cell adhesion molecules as well as the peri-tumoral matrix (Table 2) [18]. Further MMPs whose synthesis is promoted upon CD147 activation are MMP-2, MMP-9, and membrane type (MT)1-MMP (Table 2) [42,43,44], whose levels positively correlate with the size, histological grade, or stage of progression of OSCC, and are predictive of its metastatization [31,84,85,86,87].

Thus, due to its capability of activating ECM-degrading proteases, CD147 is deeply involved in the invasion of the peritumoral tissue, basement membrane, and underlying stroma by OSCC cells.

To infiltrate the tissues and move through them, cancer cells rearrange their cytoskeleton so that their plasma membrane ejects and generates protruding structures called invadopodia [44,88]. In this context, CD147 cooperates with growth factors to induce the formation of invadopodia in epithelial cells [89,90]. Of interest, invadopodia are present not only in OSCCs but also in OPMDs at high risk of neoplastic transformation [88]. In both premalignant and malignant oral lesions, CD147 is positioned on the leading edge of the invadopodia together with MT1-MMP, which, after being activated by CD147, degrades ECM both directly and by converting latent MMP-2 and MMP-9 in their active forms [26,44,91]. At the same time, CD147 stimulates CAFs to synthesize tenascin-C (Table 2) [49], an ECM molecule that facilitates OSCC cell migration [20].

In addition to CD147 and MT1-MMP, α3β1or α6β1 laminin receptors, uPA and its receptor (uPAR) are present at the invadopodia in OSCC tissue [41,48,74,92]; thus, a platform is built in which CD147 coordinates the activity of ECM-binding integrins and ECM-degrading enzymes so that cellular invasion is spatially oriented. The clinical relevance of this mechanistic model of cell invasion is supported by the finding that CD147, MT1-MMP, uPA/uPAR, α3β1, and α6β1 are expressed in OSCCs with an intensity that directly relates to the invasive and metastatic capabilities of tumors [41,48,74,92,93].

Besides mediating OSCC cell invasion of the peritumoral tissue, the concomitant activation of MMPs and uPA favors OSCC infiltration by stromal cells and leukocytes secreting cytokines and growth factors with a pro-tumor action [94,95]. However, MMPs and uPA can also facilitate the penetration of cancer tissues by immune cells endowed with antitumor activities [96,97]. Undoubtedly, the prevalence of one of these effects over the other has a great impact on the prognosis of OSCC patients. More studies are then needed to evaluate how much OSCC clinical progression is affected by the composition of immune cell infiltrate.

Once MMPs and uPA have degraded the peritumoral matrix and basement membrane, OSCC cells invade the underlying stroma, reach the lymphatic and blood vessels, adhere to their wall, penetrate it, circulate in the blood and lymph, and then arrive at new anatomical sites: there, OSCC cells extravasate and proliferate, eventually giving rise to the metastases [98]. However, while they stay in the blood or lymph, OSCC cells do not receive the survival signal that is normally provided to them by the activation of AKT and/or ERK, which follows integrins binding to a solid ECM. The lack of this signal induces in adherent cells, such as those of epithelial origin, a peculiar type of apoptosis termed “anoikis” [99]. Because of the latter, the circulating tumor cells could die, and cancer metastasization could thereby be hindered [98,99]. However, upregulated CD147 intensively activates ERK and AKT signaling (Figure 1) [61,62,63,64,65], which ultimately promotes the survival of circulating OSCC cells even in the absence of the sustainment ordinarily provided to them by a solid ECM: in doing so, CD147 favors OSCC metastatization [100,101,102].

## 3. Reciprocal Interaction between CD147 and OPMD/OSCC-Associated Inflammation

Results from clinical–epidemiological studies indicate that chronic inflammation of the oral mucosa significantly augments the risk of OSCC onset and its clinical progression [103,104,105]. Inflammation occurs in response to most of the agents causing oral lesions [27,103,104] and implies that neutrophils, lymphocytes, and monocytes extravasate in the oral tissues: this phenomenon is facilitated by the fact that leukocytes express CD147 [26,106,107], which, in turn, binds to endothelial–leukocyte adhesion molecules, such as endothelial selectin (Table 2) [51].

As is the case for oral mucositis, the number of tissue-infiltrating inflammatory cells is elevated in low-risk OPMDs as compared to healthy oral mucosa, being further augmented in high-risk OPMDs and OSCCs [105]. In these pathological settings, leucocytes release cytokines, among which interleukin (IL)-1β, IL-6, IL-8, and tumor necrosis factor (TNF)α are particularly abundant [79,108,109]. Specifically, in parallel with the increase in the number of infiltrating leukocytes, the levels of the abovementioned cytokines progressively augment as the oral mucosa becomes the site of a chronic inflammation, an OPMD, and, finally, an invasive OSCC [79,108,109]. This phenomenon has been confirmed in animal models of oral carcinogenesis [110,111]. On their part, OSCC cells constitutively express IL-1, IL-6, IL-8, and TNF-α, thereby contributing to increasing the concentration of these inflammatory mediators in the tumor tissue [112,113,114,115]. In dysplastic, hyperplastic, or neoplastic oral lesions, IL-1, IL-6, IL-8, and TNF-α could likely be produced by fibroblasts [27] or by injured keratinocytes [116,117]. The close contact between oral mucosa and saliva explains why the concentrations of IL-1, IL-6, IL-8, and TNF-α are augmented in the saliva of individuals with OPMDs compared to healthy controls and rise further in the saliva of OSCC patients [108,118,119].

The triggering of CD147 is very important to cytokine production by OSCC cells, fibroblasts, or leukocytes (Figure 1). Indeed, the synthesis of inflammatory mediators is induced or strongly increased when periodontitis-associated bacteria release the CD147 bound to the OSCC cell membrane into a diffusible form that homodimerizes with the CD147 anchored on the surface of neighbor cells, including fibroblasts, OSCC cells, and normal epithelial, endothelial, or inflammatory cells (Table 1) [27,103]. Thereafter, CD147 overstimulation turns on both the MAPK/ERK and PI3K/AKT signaling pathways leading to the activation of NF-kB transcription factor and the expression of its targeted genes (Table 2, Figure 1) [26,50,60,120,121]. Among them is the cyclooxygenase 2 (COX-2) enzyme mediating the synthesis of prostaglandin E2 [122,123], which, in turn, stimulates IL-1β, IL-6, IL-8, and TNFα production (Table 2, Figure 1) [122,124]. In agreement with these findings, the activation of CD147 is followed by the induction of inflammation and TNF-α, IL-1β, or IL-6 expression [106,125,126,127], while CD147 antagonists exert anti-inflammatory activities [128].

Normal oral keratinocytes and OSCC cells possess receptors for the above-mentioned inflammatory mediators [117,129,130,131]. Consequently, the inflammatory cytokines induced by CD147 could mutually upregulate the expression of CD147: this could occur through the activation of Fyn tyrosine kinase, which is known to induce CD147 expression in the oral mucosa (Table 1, Figure 1) [36], and it is activated by IL-1β, IL-6, and TNFα [132,133,134]. In this way, the activities of CD147 and phlogogenic mediators could fuel each other. Such crosstalk was demonstrated for IL-1 in animal models [37].

It must be highlighted that the activation of NF-kB induced by CD147 results not only in the expression of inflammatory mediators but also in the synthesis of pro-invasive MMP-2 and MMP-9 (Table 2, Figure 1) [135,136,137]. Moreover, by triggering NF-kB-promoted MMPs expression, the concerted actions of CD147 and inflammatory cytokines cause the infiltration of OPMD or OSCC tissues by neutrophils and/or monocytes [26,28,83]. While neutrophils produce and release high levels of MMP-9 [8,83], monocytes differentiate into macrophages [26]. In this context, IL-1, IL-6, and IL-8 induce the polarization of macrophages to the protumor M2 phenotype, hence accelerating OSCC progression, both in humans [138] and in animal models [104]. Indeed, M2 macrophages release into the tumor microenvironment inflammatory cytokines and growth factors that inhibit anti-tumor immune responses or promote the proliferation of neoplastic cells [139,140]. Altogether, these findings explain why the number of neutrophils or macrophages that are present in OSCC tissues directly correlates with patients’ poor clinical outcomes [94,95,138,140].

Among the molecules produced by M2 macrophages infiltrating OSCC stroma is epidermal growth factor (EGF) [140], a potent inducer of MMP expression and cellular invasiveness (Table 2) [46]. It must be highlighted that the expression of EGF receptor (EGFR) is low in the healthy oral epithelium [117], it is augmented in high-risk OPMDs [41], and it is strongly upregulated in carcinoma cells [46,89,141]. It is noteworthy that in addition to recruiting and activating EGF-producing macrophages, IL-1β, IL-6, IL-8, and TNFα increase EGFR expression and/or activity in epithelial cells (Figure 1) [142,143,144,145]. On the surface of carcinoma cells, EGFR forms a complex with CD147, which is also upregulated [46,89,141]: this leads to the phosphorylation of AKT and MAPK, which is followed by the formation of invadopodia [89]. Because of the increase in EGFR and CD147 tissue levels, the synthesis of MMP-2 and MMP-9 by OSCC cells and CAFs is augmented [41,46].

Together with EGF levels, those of Transforming Growth Factor (TGF)-β1 are increased in OSCC lesions and peritumoral tissue [141,142,143,144,145,146,147,148,149]. There, TGF-β1 is produced by both OSCC cells and CAFs [147,150]. In this framework, IL-1, IL-6, IL-8, and TNFα have been shown to stimulate the migration of specific subtypes of fibroblasts [151,152,153,154,155,156,157]. The same could occur for OSCCs, given the capability that inflammatory mediators or growth factors have to trigger CAFs locomotion [158].

In addition to OSCC cells and CAFs, also normal keratinocytes synthesize and release TGF-β1, albeit in low amounts and in a latent form [159]. The latter is activated upon its binding to αvβ6 integrin, which is not expressed in the intact oral mucosa, being induced only during the repair of oral wounds [150,159] and, in general, in inflamed tissues (Figure 1) [160].

In contrast, either αvβ6 or TGF-β1 are constitutively over-expressed by OSCC cells where αvβ6 converts latent TGF-β1 into an active form which, in turn, promotes the expression of pro-MMP-9 [150]. Thereafter, pro-MMP-9 is activated by the MT1-MMP/MMP-2 axis, triggering OSCC cell invasion [92].

Previous studies have shown that TGF-β1 receptors are poorly expressed in OSCC as compared to normal oral epithelium [41,91,161]: this finding may explain why OSCC cells are less sensitive to the effects of TGF-β1 than normal oral keratinocytes [161,162,163]. Notwithstanding, TGF-β1 effectively induces pro-MMP-9 expression by simultaneously triggering several transcriptional activators of MMPs, such as Sp1, AP-1, and NF-kB [164,165,166]. Consistently, in OSCC tissues, the overexpression of MMP-9 parallels that of TGF- β1 [146].

Regarding the inflammatory process that precedes and accompanies the onset of OPMDs and their evolution to OSCCs, it is of interest that IL-1β and IL-6 can upregulate the expression of TGF-β receptors II and I, respectively (Figure 1) [167,168].

Collectively, these findings suggest that TGF-β1 is particularly important to OSCC development, although it continues to play a role in OSCC progression as well.

Concerning CD147, both EGF and TGF-β1 upregulate its expression (Table 1) [38,39]. This is likely to depend on the fact that either growth factors trigger the MAPK and PI3K/AKT signaling pathways [169,170,171], which are known to activate the Fyn tyrosine kinase (Figure 1) [36,172].

Bidirectional crosstalk exists between CD147 and TGF-β1 in that they reciprocally induce their expression (Table 2) [57,173]. This suggests that the downregulation of CD147 occurring upon wound healing completion may turn off TGF-β1 signaling. In contrast, TGF-β1 persists at high levels in OPMDs or OSCCs, where it synergizes with EGF to trigger the CD147/MMP axis, thereby promoting an invasive phenotype in keratinocytes [174].

## 4. CD147 and Mobile Phenotype of Oral Epithelial Cells

**Figure 2:**
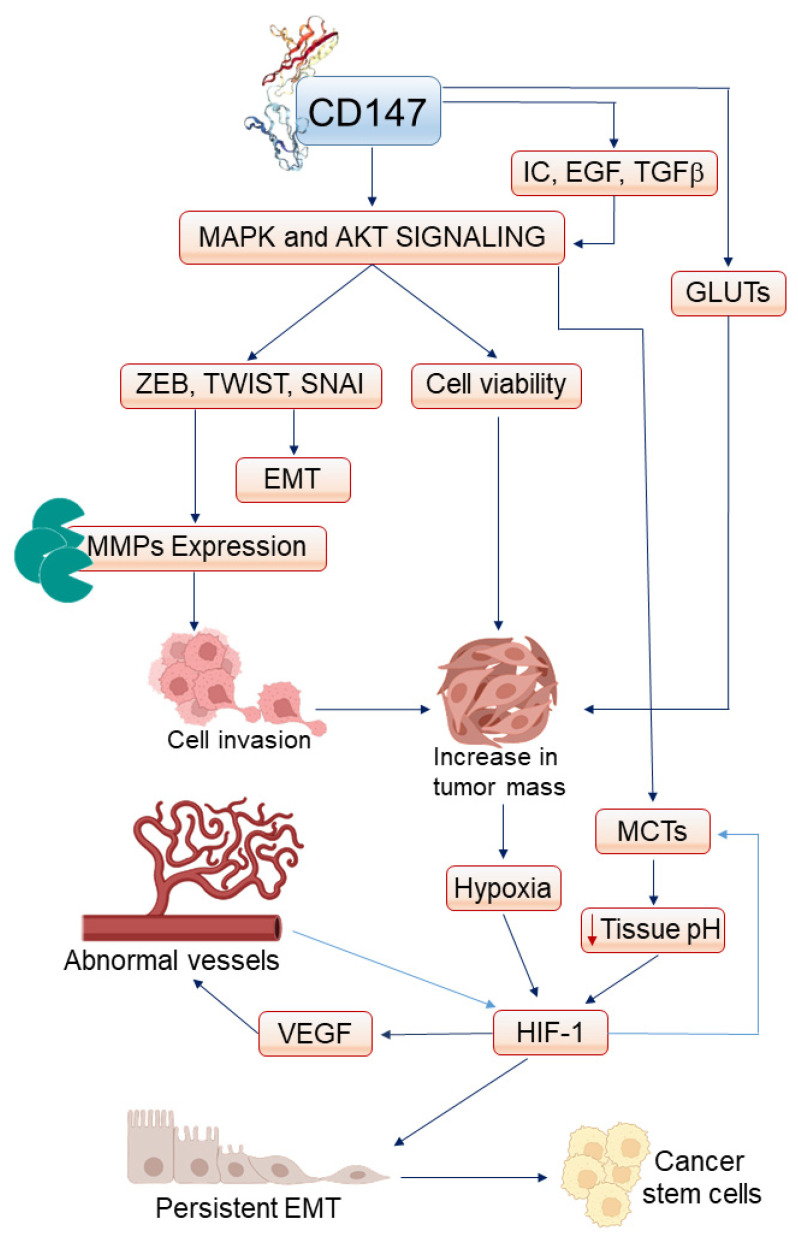
CD147 affects OSCC development and progression by modulating epithelial cell viability, growth, motility, and differentiation. Arrows symbolize directions of connections. Abbreviations: AKT, protein kinase B; EGF, epidermal growth factor; EMT, epithelial-to-mesenchymal transition; GLUT, glucose transporter; HIF-1, Hypoxia-inducible factor-1; IC, inflammatory cytokines; MAPK, mitogen-activated protein kinase; MCT, monocarboxylate transporter; MMP, matrix metalloproteinase; SNAI, zinc finger snail homolog; TGFβ, transforming growth β; TWIST, basic helix-loop-helix twist homolog VEGF, vascular endothelial growth factor; ZEB, zinc finger E-box-binding homeobox.

As discussed previously, the triggering of receptors for inflammatory cytokines and/or growth factors highly expressed in OPMDs and OSCCs is followed by the phosphorylation of the PI3K/AKT and MAPK/ERK signaling pathways, which are both overactivated in oral squamous preneoplastic and neoplastic lesions [175,176,177,178]. Upon their phosphorylation, PI3K/AKT or MAPK/ERK activate a variety of transcription factors, among which are the members of the zinc finger E-box-binding homeobox (ZEB), zinc finger snail homolog (SNAI), and/or TWIST families (Figure 2) [129,130,179,180,181,182,183,184,185].

On their part, these transcriptional activators promote epithelial-to-mesenchymal transition (EMT) (Figure 2) which is the multistep process through which epithelial cells lose their typical markers while acquiring mesenchymal cell features [146,186,187]. Specifically, in epithelial cells in which ZEB, SNAI, and/or TWIST are activated, the expression of epithelial adhesion molecules (e.g., Epithelial-cadherin) or cytoskeletal components (e.g., the cytokeratins) is repressed, while the synthesis of mesenchymal adhesion molecules (e.g., Neuronal-cadherin) or cytoskeletal components (e.g., vimentin) is induced [186,187]. As a result of the EMT process, the phenotype of epithelial cells is changed from static (that is, oriented according to an apical-basal polarity and strongly attached to the sister cells and basement membrane) to loosely connected/adhesive and mobile [170,171].

While physiological, quickly reversable EMT occurs in tissue repair, persistent and exacerbated EMT takes place during cancerogenesis [179,180,186,187]. For OSCCs and high-risk OPMDs, the concomitant presence of numerous cytokines capable of activating ZEB, SNAI, and/or TWIST induces a lasting EMT in epithelial cells, whether they are normal or transformed (Figure 2) [105,188,189]. As expectable, in either OPMDs or OSCCs, the number of EMT cells correlates with that of infiltrating inflammatory cells [105]. In agreement with the fact that the stimulation of CD147 triggers the same signaling pathways (namely MAPK/ERK and PI3K/AKT) which activate the EMT-promoting transcription factors (Figure 2), in OSCC tissues, the number of cells which have undergone EMT positively correlates with the intensity of CD147 expression (Table 2) [54,190]. Similar findings were observed in other types of human carcinomas, where CD147 upregulation and/or hyper glycosylation was accompanied by an increase in the expression and/or activity of transcription factors that induce EMT in cancer cells and eventually exacerbate their invasiveness [191,192,193]. Furthermore, infection by periodontal bacteria capable of stimulating cytokines or growth factors production by inflammatory cells is associated with the induction of EMT in oral keratinocytes [129].

Among cytokines and growth factors, TNFα and TGF-β1 are particularly effective at promoting the EMT of both normal oral keratinocytes and OSCC cells [41,130,131,146,147,148,180]. Of importance, TGF-β1 and EMT-promoting transcription factors can activate each other in a reciprocal fashion that strongly favors OSCC progression [180]. In particular, the acquisition of EMT by OSCC cells is followed by a further increase in their ability to invade the peritumor tissue, degrade the basement membrane, and penetrate the underlying stroma [41,54,131,146,148,179,190]. Furthermore, the acquisition of EMT by OSCC cells greatly favors their ability to metastasize [54]. This also occurs because EMT-promoting transcription factors can directly activate MMP expression (Table 2, Figure 2) [194,195,196]. In addition, by repressing E-cadherin expression, EMT transcription factors provoke the disassembly of E-cadherin/β-catenin complexes at the intercellular junctions: this is followed by the translocation of β-catenin from the cytoplasm to the nucleus, where β-catenin cooperates with NF-kB at inducing MMP expression [196,197]. Conversely, the knockdown of CD147 can reduce the activity of EMT-promoting transcription factors, thereby reverting the EMT and inhibiting the invasiveness of cancer cells [198].

## 5. CD147 and the Growth of OSCC

In the previous paragraphs, we described how CD147 simultaneously activates a variety of inflammatory mediators and growth factors in OPMDs and OSCCs, thereby actuating PI3K/AKT, MAPK/ERK, β-catenin, NF-kB, AP-1, Sp1, ZEB, SNAI, and Twist which, taken together, strongly upregulate MMP expression (Figure 1 and Figure 2). Such a rise in MMP levels accelerates and enlarges the degradation of interstitial and peritumoral matrices, creating new space for the proliferation and invasion of cancer cells and thereby augmenting the size of the tumor mass (Figure 2). It should also be considered that the inflammatory cytokines and growth factors whose synthesis is promoted by CD147 can directly promote OSCC proliferation [140,199,200,201]. Moreover, the survival and growth of OSCC cells are sustained by the activation of AKT and MAPK, which follows CD147 triggering (Figure 1 and Figure 2) [61,62,63,64,65]. Therefore, in addition to favoring the onset of OPMDs or their evolution to OSCCs, CD147 has an important role in increasing OSCC mass.

It is important to remember that the energy necessary for the growth of a tumor derives, for the most part, from the catabolism of glucose [202]. As seen in other types of carcinomas compared to their respective tissue of origin, OSCCs are more capable than healthy oral mucosa of taking up glucose [203]. This is because OSCC cells display glucose transporters (GLUTs) levels higher than normal oral keratinocytes [203]. Specifically, in OSCCs, GLUT-1 and GLUT-3 are overexpressed in a fashion that positively correlates with the stage of disease progression and the severity of patients’ prognosis [203]. At the plasma membrane level, CD147 interacts with the GLUTs implementing their function (Figure 2) [55]. Accordingly, CD147 overexpression parallels an increase in glycolysis [26,55] and, once again, contributes to augmenting OSCC size (Figure 2).

At variance with what occurs in normal cells, in neoplastic cells, the pyruvate that is formed upon glucose catabolism is largely converted to lactic acid [204]. Of note, the activation of the AKT/NF-kB axis that follows CD147 stimulation upregulates the expression of monocarboxylate transporters (MCTs) (Table 2, Figure 2) in carcinoma cells [56,63], a family of proteins catalyzing the cellular export of lactate [205]. The capability of CD147 to carry and concentrate both MCT1 and MCT4 on the cell membrane causes much of the lactic acid produced by cancer cells to be released by them into the tumor microenvironment, which is thereby acidified (Figure 2) [26,41]. The lowering of tissue pH inhibits the antitumor activity of cytotoxic T lymphocytes, hence further augmenting tumor growth (Table 2) [26,41].

As the neoplastic mass grows, the local blood vessels are unable to meet the increased demand for oxygen and nutrients by the proliferating tumor [206]. Consequently, in the tumor tissue, the oxygen level is reduced [206]. This condition, termed hypoxia, leads to the activation of the Hypoxia-Inducible transcription Factor (HIF)-1 (Figure 2) [206]. The latter consists of two subunits: HIF-1β, which is expressed constitutively, and HIF-1α, whose half-life is regulated by the oxygen levels [206]. Specifically, when oxygen tension is normal, HIF-1α protein is modified in a way that causes its degradation via the ubiquitin–proteasome pathway [206]. In contrast, HIF-1α is stabilized in hypoxic tissues, where it cooperates with transcriptional coactivators to induce the expression of target genes [206]. In this regard, it is noteworthy that high HIF-1α protein levels are detected in advanced, proliferating, and invasive OSCCs [207].

Among the transcriptional targets of HIF-1 is MCT-4 (Figure 2), which is highly expressed in invasive, advanced OSCCs, consistent with the activation of HIF-1 observed in the hypoxic areas of the proliferating tumor [208]. Since HIF-1 transcriptional activity is enhanced in an acidic microenvironment, HIF-1 and MCT-4 mutually amplify their functions, and this exasperates the aggressive behavior of cancer cells [41]. In fact, besides MCT-4, HIF-1 also activates EMT-promoting transcription factors, thereby further upregulating MMP expression [206]. Therefore, hypoxia, further enhances the invasive and metastatic capabilities of OSCC cells.

An additional transcriptional target of HIF is Vascular Endothelial Growth Factor (VEGF)-A (Figure 2), a powerful promoter of angiogenesis, that is the process by which new blood vessels are formed starting from pre-existing ones [206,209].

Briefly, angiogenesis implies that endothelial cells degrade the vessel basement membrane via the activity of MMPs and uPA, migrate into the perivascular space, and at the same time proliferate, forming cellular cords: eventually, the latter cavitate, allowing blood outflow from the pre-existing vessel to the newly formed one [209]. In healthy adults, these events mostly take place during tissue repair [209]. In contrast, an abnormal angiogenesis accompanies tumor progression, when the new vessels are required to meet the increased demand for oxygen and nutrients by the proliferating cells [209]. As for other types of premalignant lesions, the number of vessels supplying OPMDs with a high risk of neoplastic transformation is generally higher than that present in the connective tissue underlying healthy oral mucosa [31]. Vessel density increases further in OSCCs, where it correlates positively with a low index of differentiation and/or with a high metastatic potential of the tumor [210,211], the second feature being consistent with the fact that the newly formed vessels also constitute new routes through which cancer cells may spread throughout the body [209]. The stimulation of angiogenesis occurring in high-risk OPMDs and OSCCs is paralleled by the concomitant overexpression of CD147, MMP-9, and VEGF [38,81,211]. In fact, as is the case with MMP-9, the triggering of CD147 in cancer cells and peritumoral fibroblasts leads to the synthesis of VEGF (Table 2), which is preceded by PI3K/AKT and MAPK signaling (Figure 2) [53]. Thus, CD147 cooperates with HIF-1 to promote VEGF expression. This is confirmed by clinical findings indicating that VEGF production is very abundant in neoplastic tissues where CD147 is upregulated and MAPK and PI3K/AKT are activated [53]. These events may help to explain why high levels of the CD147 agonist cyclophilin A detectable in OSCC tissues are mirrored by elevated VEGF levels [74].

## 6. CD147 and OSCC Resistance to Therapy

Although they can sustain OSCC growth, tumor vessels are malformed and poorly functioning: this causes hypoxia to persist in neoplastic tissues [212,213], leading to chronic activation of HIF-1, which, in turn, promotes an enduring EMT (Figure 2) [214]. This phenomenon is associated with the emergence of OSCC lesions of cells which are so dedifferentiated that they resemble stem cells (Figure 2) [189]. These cells are found next to the cells that have undergone EMT and indeed express stem markers including CD133, Notch, and aldehyde dehydrogenase 1 [189,215,216,217,218,219]. Many of the stem-like cells populating OSCC tissues are cancerous (cancer stem cells) [41], and their number is negatively correlated with OSCC differentiation grade [215].

Normally, stem cells reside in the basal layer of the oral epithelium, from where they migrate to the superficial layers, and then differentiate into the mature epithelial cells that replace apoptotic epithelial cells [41]. Oral epithelial stem cells express high levels of cytoplasmic β-catenin together with surface markers including CD147, E-cadherin, and CD44 [41]. The latter is a transmembrane glycoprotein whose extracellular portion binds to the glycosaminoglycans of ECM, promoting cell survival via the activation of AKT signaling [220].

In healthy oral epithelium, CD44 and CD147 expressed by stem cells of the basal layer cooperate in wound healing [41].

At variance with normal basal stem cells, the stem-like cells present in OSCCs display low E-cadherin/β-catenin and high CD147/CD44 levels [41]. The concomitant overexpression of CD147 and CD44 makes the cancer stem cells present in OSCCs very viable and invasive [141,215,216,217,218,219,220,221]. In fact, while CD147 triggers MMP-9 expression, CD44 anchors MMP-9 in the invadopodia, thus enforcing and orienting cellular invasion [41]. Moreover, the concurrent overactivation of CD147 and CD44 strongly sparks AKT pro-survival signaling, protecting circulating cancer cells from anoikis [41] and strengthening their resistance to chemotherapy and/or radiotherapy [33,222,223,224]. Furthermore, CD44 stimulation upregulates the expression of multidrug transporters and multidrug resistance-associated proteins [41]. Accordingly, OSCCs rich in cancer stem cells are characterized by a high rate of relapse and metastasis and poor sensitivity to antitumor therapies [215,216,217,218,219,220,221].

In addition to being implicated in OSCC clinical progression, cancer stem cells are also likely to be involved in OSCC onset [221], as reported for other types of SCC, which originate from the malignant transformation of the stem cells of epithelium basal layers [225]. Nonetheless, carcinoma may also result from the dedifferentiation and subsequent transformation of mature epithelial cells [226,227,228,229]. As for OSCC, both occurrences are likely to contribute to the onset of cancer. Indeed, cell dedifferentiation and cell transformation are induced by common mechanisms, such as the inactivation of tumor suppressor genes and/or the mutation of protooncogenes into oncogenes [230]. Some of these molecular events are sparked by pathogens promoting the development of OPMDs and their progression into OSCC [229,231].

## 7. Conclusions and Future Perspectives

The incidence of OSCC, already high in previous decades, is still increasing at present, causing a considerable number of deaths [1,2,3]. In this regard, it should be considered that although the oral cavity is easily inspected, patients only come to the surgeon when the tumor is fully symptomatic, that is, when it is in an advanced stage of progression [1,2,3]. At that point, the OSCC requires demolishing and disabling surgery which cannot prevent the relapse or metastatization of the tumor [5]. In addition, late stage OSCC is often weakly sensitive or even resistant to chemotherapy and/or radiotherapy [5,6]. Therefore, the timeliness of OSCC diagnostic assessment makes a difference for patients, often saving their lives.

Results from in vitro, animal, and clinical studies candidate CD147 as a reliable marker to be used for the early diagnosis of OSCC. Indeed, the levels of CD147 in the oral mucosa increase when the latter is exposed to pro-inflammatory pathogens, which play an important role in OSCC onset [29]. CD147 expression is further upregulated in OPMDs, especially in those at high risk of neoplastic transformation [18,19,30,31]. Finally, CD147 is expressed at very high levels in OSCCs, where it is activated to the extent that directly relates to tumor capability of relapsing and/or metastasizing [18,19,30,31]. Most noticeably, the reciprocal interactions among CD147 and the inflammation or hypoxia that accompany OSCC progression cause keratinocytes to acquire EMT or stemness features [41,54,130,131,146,148,190,215], which increase cellular invasiveness and resistance to apoptosis [186,187].

The graduality with which CD147 levels increase during the sequential steps of oral carcinogenesis is mirrored by the progressive activation of CD147-stimulated intracellular signaling pathways [27,43,53,60,61,62,63,64]. In particular, the phosphorylation of AKT triggered by CD147 sparks molecular and cellular events, which are key to both the development and clinical progression of OSCC (Table 3).

Among these events is the production of pro-invasive MMPs [232] and the activation of the NF-kB/COX-2/prostaglandin E axis with the consequent expression of EMT-promoting inflammatory mediators (Figure 1 and Figure 2) [120]. A further effect resulting from CD147-promoted AKT phosphorylation is an increase in glucose uptake and lactate secretion by OSCC cells (Figure 2) [234]. The increase in glucose uptake is very likely to be involved in the tumor growth effect promoted by the stimulation of the CD147/AKT axis [235], while the acidification of the tumor microenvironment, which results from lactate secretion, favors HIF-1 activation that, in turn, triggers angiogenesis [63,206] and contributes to the induction of cell dedifferentiation (Figure 2) [189,214,215,216,217,218,219]. The development of angiogenesis is accelerated by the fact that CD147-promoted AKT phosphorylation directly stimulates VEGF synthesis (Figure 2) [53]. Finally, the activation of AKT triggered by CD147 augments the viability of cancer cells [223,224,233]. Consequently, tumor cells survive anoikis, favoring cancer spreading and metastatization and resistance to chemotherapy or radiotherapy (Table 3) [41,223,224,233].

Taken together, these findings not only strongly support the use of CD147 as a diagnostic–prognostic marker for OSCC but also encourage the evaluation of CD147 as a target of anti-OSCC innovative therapies, hopefully, more effective than the conventional ones used so far.

In fact, in accordance with the fact that CD147 sparks AKT, which, in turn, provides OSCC cells with a survival signal that renders them resistant to cytotoxic drugs, CD147 antagonists increase OSCC cells’ sensitivity to fluorouracil [236]. Still, regarding CD147-promoted AKT activation, it must be highlighted that blocking AKT phosphorylation reduces the proliferation of OSCC cells and induces their apoptosis [100,101]. Moreover, AKT antagonists downregulate the expression of CD147 [237], as well of its targets, including COX-2, IL-6, TNF-α, or MMP-9 [238].

A new protocol for the treatment of OSCC could therefore provide that conventional anticancer chemotherapy and/or radiotherapy are supplemented by antagonists of the CD147/AKT axis. These could possibly be accompanied by COX-2 inhibitors, which may further reduce the synthesis of OSCC developmental/progression factors by blocking NF-kB transcriptional activity [22,239,240].

## Figures and Tables

**Table 1:** CD147 expression and/or activity are stimulated by a wide variety of molecules, including CD147 itself.

CD147 Stimulator	Action	Reference
Fyn tyrosin kinase	Triggering CD147 expression	Ramos DM et al. [36]
Interleukin-1	Induction of CD147 expression	Wang Q et al. [37]
Epidermal Growth Factor	Upregulation of CD147 expression	Omi Y et al. [38]
Transforming Growth Factor-β1	Upregulation of CD147 expression	Wang W et al. [39]
Glycosyltransferases	CD147 activation	Bai Y et al. [32]
CD147 anchored to the surface of (or released by) neighboring cells	CD147 activation	Guindolet D et al. [26]
Galectin 3	CD147 activation	Mauris J et al. [28]
Cyclophilin A	CD147 activation	Takahashi M et al. [33]

**Table 2:** CD147 modulates the expression or function of transcriptional activators, cytokines, and proteolytic enzymes with a role in the development and/or progression of OSCC.

CD147-Targeted Molecule	Effect of the Action Carried Out by CD147	References
α3β1, α6β1	basal epithelial cells’ adhesion to the basement membrane	Richard V et al. [41]
MMP-1	disruption of intercellular adhesion, epithelial cell locomotion and growth	Cao Z et al. [18]
MT1-MMP	ECM degradation, MMP-2 or -9 activation, cellular invasion	Mitre GP et al. [44]
MMP-2	ECM degradation, cellular invasion	Luo Z et al. [42]
MMP-9	ECM degradation, cellular invasion	Suzuki S et al. [43]
TIMPs	increase in MMPs activity	Maghsood F et al. [47]
uPA	ECM degradation, plasminogen or MMPs activation, cellular invasion	Lescaille G et al. [48]
Tenascin	facilitation of OSCC cell migration	Dang D et al. [49]
NF-kB	induction of COX-2, inflammatory cytokines, and MMPs expression	Yu B et al. [50]
Endothelial selectin	leukocytes extravasation	Muramatsu T [51]
EGF and TGF-β1	EMT and cell invasion	Wu J et al. [52]
VEGF	Angiogenesis	Tang Y et al. [53]
ZEB, SNAI, TWIST	EMT, MMPs expression	Siu A et al. [54]
GLUTs	increase in glucose uptake by OSCC cells	Almeida LMCA et al. [55]
MCTs	lactate export from OSCC cells, functional impairment of CD8^+^ T cells, HIF-1 activation	Kirk P et al. [56]
HIF-1	MMPs or VEGF expression, cell invasion, angiogenesis	Wang CH et al. [57]
CD44	survival, anchorage-independent growth, and drug resistance of OSCC cells	Richard V et al. [41]

**Table 3:** Effects directly resulting from AKT activation promoted by CD147 stimulation.

Effect	Consequence	Reference
Synthesis of MMPs	Cell invasion	Ding P et al. [232]
Activation of the NF-kB/COX-2 axis	Inflammatory cytokines expression, EMT	Dana P et al. [120]
Upregulation of MCTs	Lowering of tissue pH, activation of HIF-1	Dana P et al. [63]
Synthesis of VEGF	Angiogenesis	Tang Y et al. [53]
Cell survival—I	Circulating cancer cells escape anoikis	Ke X et al. [233]
Cell survival—II	Cancer cells resist chemotherapy	Kang MJ et al. [223]
Cell survival—III	Cancer cells resist radiotherapy	Wu J et al. [224]

## Data Availability

Not applicable.

## Abbreviations

AKT	protein kinase B
AP	activator protein
CAF	cancer-associated fibroblast
CD	cluster of differentiation
COX	Cyclooxygenase
ECM	extracellular matrix
EGF	epidermal growth factor
EGFR	epidermal growth factor receptor
EMT	epithelial-to-mesenchymal transition
ERK	extracellular-regulated kinase
GLUT	glucose transporter
HIF	Hypoxia-Inducible Factor
IL	Interleukin
MAPK	mitogen-activated protein kinase
MCT	monocarboxylate transporter
MMP	matrix metalloproteinase
MT-MP	membrane type-matrix metalloproteinase
NF-kB	Nuclear Factor-kappa B
OPMD	oral potentially malignant disorder
PI3K	phosphoinositide 3 kinase
SCC	squamous cell carcinoma
SNAI	zinc finger snail homolog
Sp	Specificity protein
TGF	transforming growth factor
TIMP	tissue inhibitor of matrix metalloproteinase
TNF	tumor necrosis factor
TWIST	basic helix-loop-helix twist homolog
uPA	urokinase-type plasminogen activator
uPAR	urokinase-type plasminogen activator receptor
VEGF	vascular endothelial growth factor
ZEB	zinc finger E-box-binding homeobox
